# The ER-Bound RING Finger Protein 5 (RNF5/RMA1) Causes Degenerative Myopathy in Transgenic Mice and Is Deregulated in Inclusion Body Myositis

**DOI:** 10.1371/journal.pone.0001609

**Published:** 2008-02-13

**Authors:** Agnès Delaunay, Kenneth D. Bromberg, Yukiko Hayashi, Massimiliano Mirabella, Denise Burch, Brian Kirkwood, Carlo Serra, May C. Malicdan, Andrew P. Mizisin, Roberta Morosetti, Aldobrando Broccolini, Ling T. Guo, Stephen N. Jones, Sergio A. Lira, Pier Lorenzo Puri, G. Diane Shelton, Ze'ev Ronai

**Affiliations:** 1 Signal Transduction, The Burnham Institute for Medical Research, La Jolla, California, United States of America; 2 Department of Pharmacology and Systems Therapeutics, Mount Sinai School of Medicine, New York, New York, United States of America; 3 National Institute of Neuroscience, Tokyo, Japan; 4 Department of Neuroscience, Catholic University, Rome, Italy; 5 Department of Pathology, School of Medicine, University of California San Diego, La Jolla, California, United States of America; 6 Department of Cell Biology, University of Massachusetts Medical School, Worcester, Massachusetts, United States of America; 7 Immunobiology Center, Mount Sinai School of Medicine, New York, New York, United States of America; 8 Dulbecco Telethon Institute at Fondazione European Brain Research Institute (EBRI)/S.Lucia 00134, Rome, Italy; University of Hong Kong, China

## Abstract

Growing evidence supports the importance of ubiquitin ligases in the pathogenesis of muscular disorders, although underlying mechanisms remain largely elusive. Here we show that the expression of RNF5 (aka RMA1), an ER-anchored RING finger E3 ligase implicated in muscle organization and in recognition and processing of malfolded proteins, is elevated and mislocalized to cytoplasmic aggregates in biopsies from patients suffering from sporadic-Inclusion Body Myositis (sIBM). Consistent with these findings, an animal model for hereditary IBM (hIBM), but not their control littermates, revealed deregulated expression of RNF5. Further studies for the role of *RNF5* in the pathogenesis of s-IBM and more generally in muscle physiology were performed using *RNF5* transgenic and KO animals. Transgenic mice carrying inducible expression of RNF5, under control of β-actin or muscle specific promoter, exhibit an early onset of muscle wasting, muscle degeneration and extensive fiber regeneration. Prolonged expression of RNF5 in the muscle also results in the formation of fibers containing congophilic material, blue-rimmed vacuoles and inclusion bodies. These phenotypes were associated with altered expression and activity of ER chaperones, characteristic of myodegenerative diseases such as s-IBM. Conversely, muscle regeneration and induction of ER stress markers were delayed in RNF5 KO mice subjected to cardiotoxin treatment. While supporting a role for RNF5 Tg mice as model for s-IBM, our study also establishes the importance of RNF5 in muscle physiology and its deregulation in ER stress associated muscular disorders.

## Introduction

Skeletal muscles are continually subjected to remodeling as a consequence of normal mechanical or metabolic stress, as an adjustment of muscle mass to muscle load during normal activity, or as a response to a muscle disease. While exercise leads to increased protein synthesis and build-up of muscle mass (hypertrophy), disuse is associated with degradation of muscle components (atrophy) [1], [2]. Atrophy can also occur during normal aging and as a result of pathological conditions such as primary muscle or peripheral nerve disease, cachexia and cancer [3]. During muscle remodeling, changes in protein turnover due to controlled degradation are balanced by new protein synthesis.

The ubiquitin-proteasome system (UPS) is a key player in muscle dynamics both in normal and in pathological conditions. The UPS is able to selectively target for degradation structural and regulatory components following their ubiquitination by different E3 ligases. Increased general UPS components, and in particular specific E3 ligases, have been implicated in muscle remodeling [2], [4], [5]. The important role of UPS in muscle atrophy is exemplified by MuRF1, a RING finger E3 ligase, and MAFbx, an F-Box protein component of the Skp1-Cullin F-Box protein (SCF) complex [6]. Increased expression of these ligases in mouse models of muscle disuse results in degradation of structural components of the muscle fibers such as the myofibrillar proteins (myosin, titin, troponin, nebulin, myotilin; [7] [8]. Conversely, induced muscle atrophy is prevented in MuRF1 and MAFbx Knockout (KO) mice [6], [7]. The E3 ligase MuRF3 has also been shown to elicit a protective effect on myocardial function through its action on two types of filamins involved in muscle mechanosensing and signaling [9].

Other E3 ubiquitin ligases, such as ZNF216, have been implicated in general muscle physiology and muscle atrophy [10]. Ozz-E3 was identified as an E3 ligase that targets sarcolemma-associated β-catenin for degradation and controls growth and maintenance of myofibrils [11]. A similar role in myofibrillogenesis has been proposed for the MuRF2 E3 ligase [12]. These studies support the notion that E3 ligases are an integral component of muscle dynamics during maintenance of normal muscle function.

In muscle disease, recent evidence supports a role of the UPS in the pathogenesis of select myopathies. Mutations in TRIM32 RING finger E3 ligase have been associated with Limb-Girdle Muscular Dystrophy (LGMD) type 2H [13] and sarcotubular myopathy [14]. Although the mechanism linking TRIM32 ligase activity to LGMD type 2H is unknown, TRIM32 has been shown to interact with myosin and to ubiquitinate actin [15], suggesting a role in myofibrillar turnover during muscle adaptation. Accumulation of ubiquitin-containing cytoplasmic aggregates has been associated with both sporadic and hereditary forms of inclusion-body myositis (sIBM and hIBM). These findings suggest an important role of the UPS in accumulation, misfolding and aggregation of muscle proteins [16]. This has been confirmed by genetic and histopathological analysis that links IBM-like muscle disorders with impairment of the endoplasmic reticulum associated degradation (ERAD) machinery, a degradation pathway important in removal of misfolded proteins [17].

The sporadic form of IBM (sIBM) is the most common acquired myopathy that occurs in the older human population. sIBM is a slowly progressive myopathy that combines both a T-cell mediated autoimmune inflammatory response and myodegenerative features including vacuolar degeneration, protein aggregation and inclusion formation [16]. Remarkably, sIBM patients are poorly responsive to anti-inflammatory or immunosuppressive treatments, suggesting that inflammation per se may not be a primary cause of the disease [16]. Among current hypotheses, accumulation of malfolded proteins such as amyloid β Precursor Protein (aβPP) and its processed forms, and ER overload associated with proteasomal dysfunction are thought to be key players in sIBM pathogenesis [16]. This hypothesis has been further substantiated by the fact that different ER stress markers, such as GRP78 and GRP94 are overexpressed and present in amyloid containing aggregates in sIBM muscles [16], [17], most likely as a consequence of the activation of the Unfolded Protein Response (UPR). Therefore, ER stress induction and impaired clearance of malfolded proteins are thought to be part of the pathogenic process occurring in s-IBM.

We have previously shown that the RING finger ubiquitin ligase RNF5 (also known as RMA-1; [18], [19]) plays an important role in muscle physiology using the *C. elegans* model [20]. RNF5 is a membrane-bound E3 ligase that is conserved from worm to human. In *C. elegans,* RNF-5 localizes to the dense bodies and the M-line of the body wall muscles, where it regulates the levels of the LIM domain protein UNC-95. In *C. elegans*, UNC-95 has been genetically associated with uncoordinated movement [21] and shown to be important for formation of muscle attachment sites (M-lines and Z-lines) associated with the downstream process of sarcomere organization [20]. In a mammalian cell culture system, RNF5 has been shown to ubiquitinate and regulate the localization of the protein paxillin [22], a critical component of focal adhesion, involved in cell adhesion and motility. More recently, RNF5 has been described as a new component of the ERAD machinery, where it contributes to the ubiquitination-dependent degradation of malfolded proteins as part of cell protein quality control [23].

Here, we have established and characterized inducible transgenic mouse over-expressing RNF5 either ubiquitously or in a muscle specific manner. We show that general as well as tissue specific overexpression of RNF5 induces myofiber degeneration associated with an alteration of endoplasmic reticulum (ER) function. Conversely, RNF5 KO mice exhibit delayed repair of muscle damage associated with attenuated ER stress response. Screening for RNF5 expression in muscle biopsies from patients suffering from muscular disorders including classical myopathies such as Duchenne and Becker myopathy as well as other myopathies with unknown etiology identified upregulation and mislocalization of RNF5 to aggregates in muscles from sIBM patients, as well as in a mouse model for hereditary IBM. Our findings establishes the importance of RNF5 in muscle physiology and in ER stress associated muscular disorders while pointing to the possible use of *RNF5* transgenic mouse as a unique model to study the role of ER function in the pathogenesis of degenerative muscle diseases.

## Results

### Aberrant RNF5 expression in human myopathies

**Figure 1:**
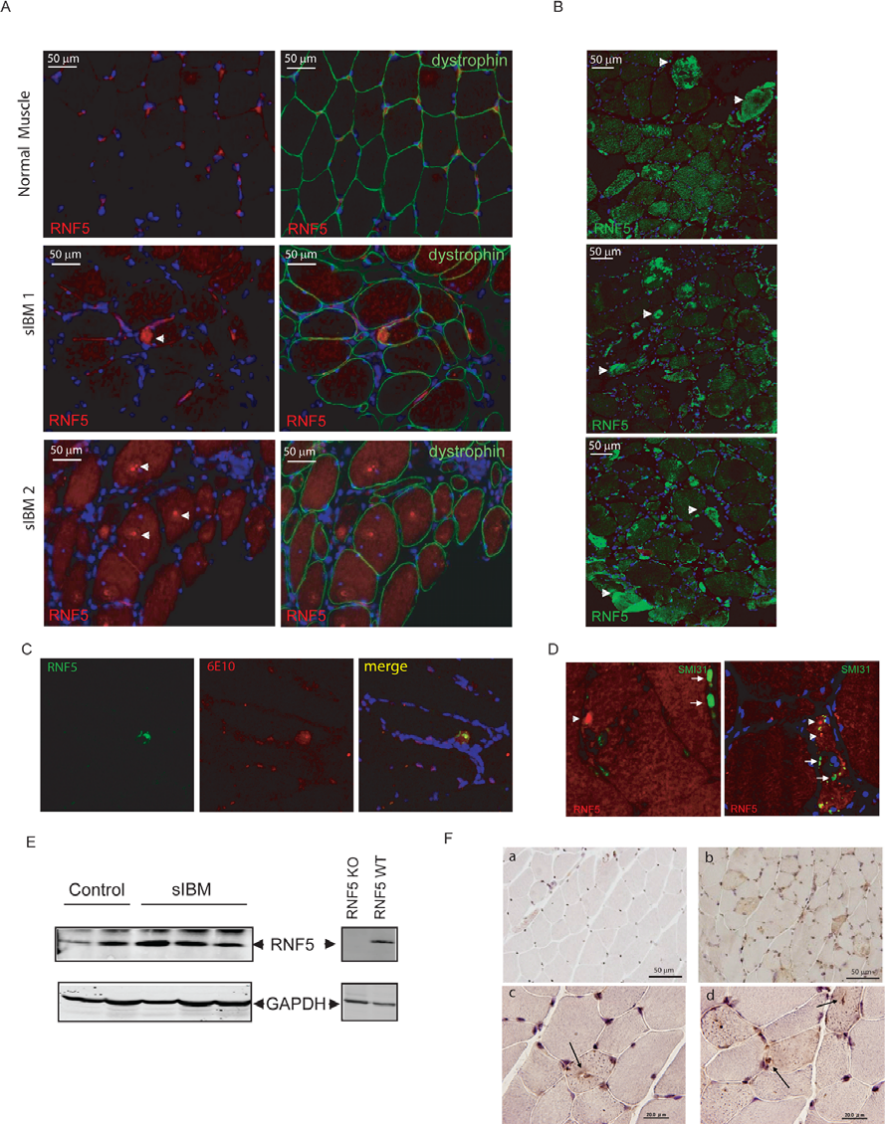
Alteration of RNF5 pattern is evident in muscle biopsies from both patients with sIBM and in a hIBM mouse model. A. Immunohistological analysis of RNF5 protein in a normal muscle biopsy and in biopsies from 2 representative sIBM patients. RNF5 staining was also positive in aggregates from 10 independent sIBM patients (not shown). B. Muscle cross-sections from 3 sIBM patients immunostained with RNF5 antibody exhibiting high levels of RNF5 protein in giant aggregates. C. Co-localization of RNF5 with beta-amyloid protein (6E10) inclusions in a representative muscle cross-section from a sIBM patient. D. Co-staining of muscle sections from 2 sIBM patients with RNF5 and SMI31, a marker for phospho-tau protein. Arrows point to phospho-tau containing aggregates and arrowheads to RNF5 staining. E. Elevated RNF5 protein levels in biopsies from 3 IBM patients. RNF5 was immunoprecipitated from equal protein amounts prepared from biopsies of sIBM patients using RNF5 polyclonal antibody. The resulting immunoprecipitates were analyzed by western blot using RNF5 antibody. Equal volumes of supernatants from the respective immunoprecipitations were analyzed for GAPDH expression, confirming that the immunoprecipitation reactions were performed using comparable amounts of extracts. F. Immunohistological analysis of RNF5 protein in control (a) and DMRV mutant mice (b–d). c and d represent higher magnification and highlight the presence of dense intrafiber staining for RNF5 in muscle fibers (arrows on panel c point to RNF5 staining within rimmed vacuoles [24] whereas arrow on panel d points to RNF5 staining within centrally located nuclei).

To assess whether RNF5 expression or localization would be deregulated in certain human myopathies associated with ER impairment, muscle biopsies from patients affected by different forms of myopathies were screened for possible changes in pattern or level of RNF5 expression using RNF5 antibodies with confirmed specificity developed in our laboratory (Fig. S1). An alteration of RNF5 expression in both vacuolated muscle fibers and in apparently normal fibers (2–20% of total fibers) was detected in biopsies from 10 patients with sIBM. Compared to control muscle, RNF5 in biopsies from sIBM patients was increased in amount and mislocalized, most frequently in apparently non vacuolated muscle fibers and diffusely within the cytoplasm (Fig. 1A). Strikingly, in 3 out of 10 biopsies from sIBM patients, intensive staining for RNF5 was evident with formation of giant aggregates inside some muscle fibers (Fig. 1B). Interestingly, RNF5 inclusions partly co-localized with beta-amyloid positive aggregates (Fig. 1C) but not with phospho-tau containing structures (Fig. 1D). Given the limited expression of RNF5 in skeletal muscle, endogenous RNF5 was not detectable by straight western blots in extracts from human biopsies. Elevated RNF5 protein levels could be observed after RNF5 immunoprecipitation in biopsies from sIBM patients (Fig. 1E). The specificity of the immunoprecipitation reaction was confirmed using muscle extracts from RNF5 KO and WT mice (Fig. 1E right panel). Unlike in sIBM, common muscular dystrophies associated with mutations in proteins of the dystrophin glycoprotein complex (DGC) such as Duchenne or Becker forms of muscular dystrophy, did not exhibit any alteration in the pattern or amount of RNF5 expression (Fig. S2). Since beta-amyloid accumulation and impairment of ERAD and UPR functions are thought to be upstream events in sIBM development [16], our data points to RNF5 deregulation as an early event in the onset of the disease.

To determine whether changes in amount or localization of RNF5 was a common occurrence in IBM type degenerative myopathies, additional analysis was performed in a mouse model of hereditary IBM, the distal myopathy with rimmed vacuoles (DMRV) mutant mouse [24]. Transgenic animals, but not their control littermates, exhibited a strong increase in RNF5 expression in muscle fibers within areas of regeneration including rimmed vacuoles and centrally located nuclei (Fig. 1F), characteristic of this model [24].These findings reveal altered RNF5 expression in specific degenerative myopathies associated with dysfunction of the ER or the ERAD machinery such as sporadic and hereditary IBM.

### Construction of RNF5 transgenic mouse

**Figure 2:**
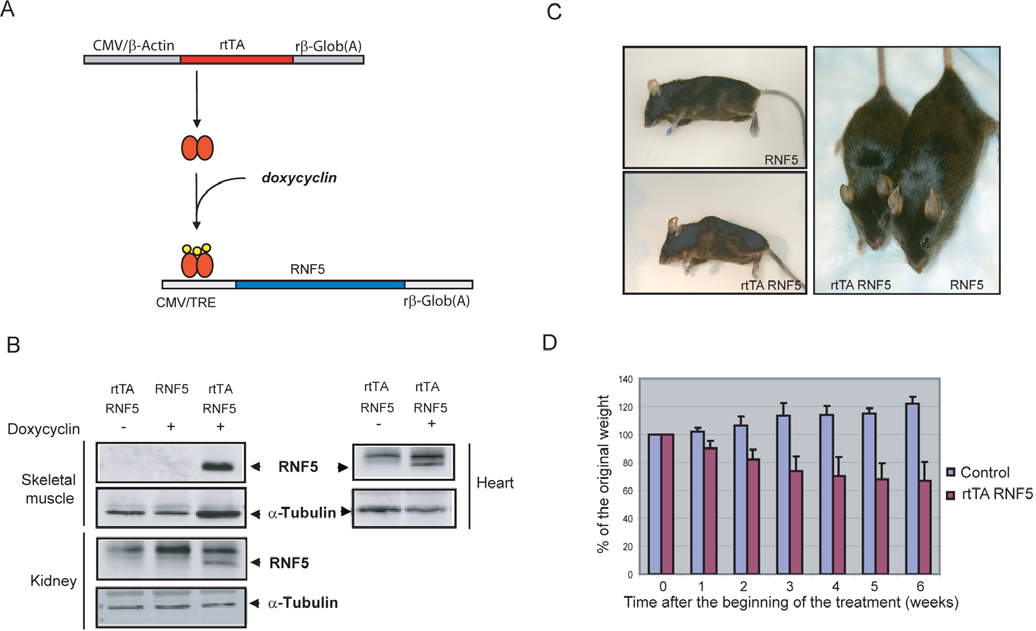
Conditional expression of *RNF5* in a transgenic mouse system. A Schematics depicting transgenic constructs used to overexpress *RNF5* in mice. The *rtTA* transcriptional activator is expressed under the control of a CMV enhancer/chicken β-actin promoter. The *RNF5* transgene is expressed under the control of a tetracycline-responsive promoter and is activated only in the presence of both the *rtTA* activator and doxycyclin. B. Tissue expression of RNF5 transgene in double transgenic animals. *rtTA RNF5 DTg* animals were treated with 2 mg/ml of doxycyclin in drinking water for 10 days and RNF5 protein levels were monitored in different organs. RNF5 expression was assessed using RNF5 polyclonal antibody, either by western blotting (skeletal muscle) or after immunoprecipitation (heart, kidney). α-tubulin was used as a loading control. In the case of heart and kidney, equal volumes of supernatants obtained following the immunoprecipitation were loaded and probed with α-tubulin. C. Comparison of a representative *DTg* animal overexpressing RNF5 after 4 wk of doxycyclin treatment with its control littermate. D. Weight curve comparison of RNF5-overexpressing *DTg* animals and their matching controls during doxycyclin treatment. The body mass of individual animals was monitored on a weekly basis for 6 wk. Graphs represent the change in body mass relative to the original weight of a single animal (n = 5). Note: In the same gender and age class, both experimental and control animals had the same external appearance and similar weight at the beginning of treatment.

In order to address the function of RNF5 deregulation in degenerative myopathies, we constructed a transgenic mouse model which allowed conditional RNF5 overexpression. A construct containing the *RNF5* gene was cloned downstream of the minimal Tet-ON operator (Tetracyclin Responsive Element (TRE)-containing promotor). *RNF5* transgenic (Tg) lines were then established by pronuclear injection and implantation in C57/BL6 recipient mice (see Methods for details). The *rtTA RNF5* double transgenic animals (*DTg*) and their corresponding control littermates (*RNF5 STg*) were generated by crossing *RNF5 STg* with *rtTA* animals, expressing the tetracyclin responsive Transcriptional Activator under the control of the ubiquitous CMV-β-actin promotor (Fig. 2A; [25].

Overexpression of RNF5 protein was confirmed in different organs of the *rtTA RNF5 DTg* animals provided with doxycyclin in drinking water (Fig. 2B). Expression of RNF5 protein was evident in double transgenic animals, but not in doxycyclin-treated single transgenic animals (*RNF5 STg*) or untreated *DTg* animals. The *RNF5* transgene is expressed at different levels depending on the organs analyzed. The higher level of RNF5 expression was seen in skeletal muscle where RNF5 transgene could be detected by straight western blot. RNF5 was expressed to a lesser extent in heart and in kidney where immunoprecipitation was required to detect the protein (Fig. 2B). Conversely, RNF5 levels were very low in liver and undetectable in brain, lungs and spleen (data not shown). This expression pattern is consistent with the low transcriptional levels of the rtTA activator and the differential expression pattern described for the rtTA transgene [25]. These data suggest that RNF5 expression is tightly controlled in *DTg* mice and that the system is not subjected to transcriptional leakage in the absence of doxycyclin induction. Furthermore, the expression of RNF5 in skeletal muscles makes it a suitable system for studying its function in this organ, without restricting its expression to mature muscle fibers.

### Induction of RNF5 transgene leads to rapid weight loss and early onset of muscle wasting and kyphosis

*rtTA RNF5 DTg* but not control animals subjected to doxycyclin treatment exhibited a significant weight loss as early as 2 wk post-induction (Figs. 2C, 2D). By 4 wk, clear phenotypic differences were evident between the double transgenic animals and their control littermates, including a significant decrease in body mass and visible kyphosis (Fig. 2C, 2D). After 5–6 wk of RNF5 overexpression, the phenotype was even more severe: the animals showed decreased activity and pelvic limb weakness. Death eventually occurred 7 weeks following initiation of doxycyclin treatment.

**Figure 3:**
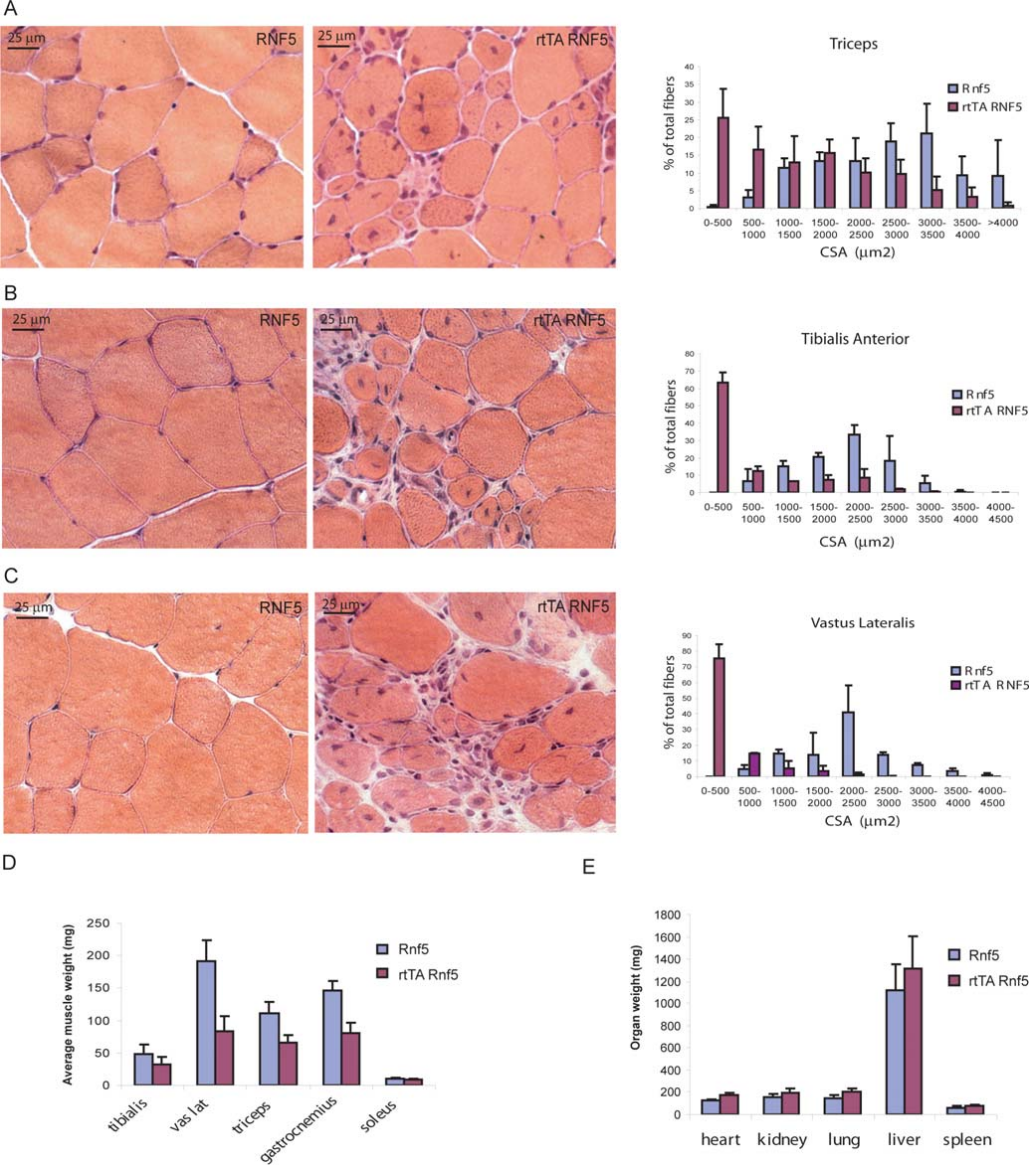
RNF5-overexpression is associated with muscle fiber degeneration. A–C Cross-sections of fresh frozen specimens of triceps brachii (A), tibialis anterior (B) and vastus lateralis (C) muscles from *RNF5 STg* or *rtTA RNF5 DTg* animals treated with doxycyclin for 6 wk (H&E stain). Fiber cross-sectional area (CSA µm^2^) corresponding to each muscle type was calculated and plotted as a percentage of the total number of fibers analyzed (n>200) (right panel). D. Average muscle mass of *RNF5 STg* and *rtTA RNF5 DTg* animals treated with doxycyclin for 6 wk. Each muscle was extracted, trimmed under the microscope and weighed on a precision scale (n = 5 for experimental and control groups). E. Average organ mass of *RNF5 STg* and *rtTA RNF5 DTg* animals treated with doxycyclin for 6 wk.

Skeletal muscles were collected from double transgenic mice and analyzed for histopathologic changes. Compared with controls, double transgenic animals exhibit clear pathological changes in all skeletal muscles analyzed (Fig. 3A–C). In transverse sections of fresh frozen specimens from the triceps brachii, tibialis anterior and vastus lateralis muscles, there was a marked variability in myofiber size in the treated double transgenic animals, with numerous small caliber fibers having a round shape, and multifocal clusters of mononuclear cells. As there was no elevation of atrogin-1 in the muscle from the double transgenic animals, the decrease in fiber size was not likely a result of muscle atrophy (data not shown). Many muscle fibers of both small and larger caliber contained internal nuclei. Measurement of the cross-sectional area confirmed the presence of many small fibers, most markedly within the vastus lateralis muscle (Fig. 3C). The small size of the muscle fibers was reflected by a decrease in the weight of each muscle (Fig. 3D). Loss of muscle mass may account for the global decrease in body weight observed in the double transgenic animals, as the weight of the other organs was not affected (Fig. 3E). Of the different muscles analyzed, the vastus lateralis was the most affected, demonstrated both by decreased cross-sectional area measurement and more pronounced weight loss.

### RNF5 overexpression is associated with increased muscle degeneration and extensive regeneration

**Figure 4:**
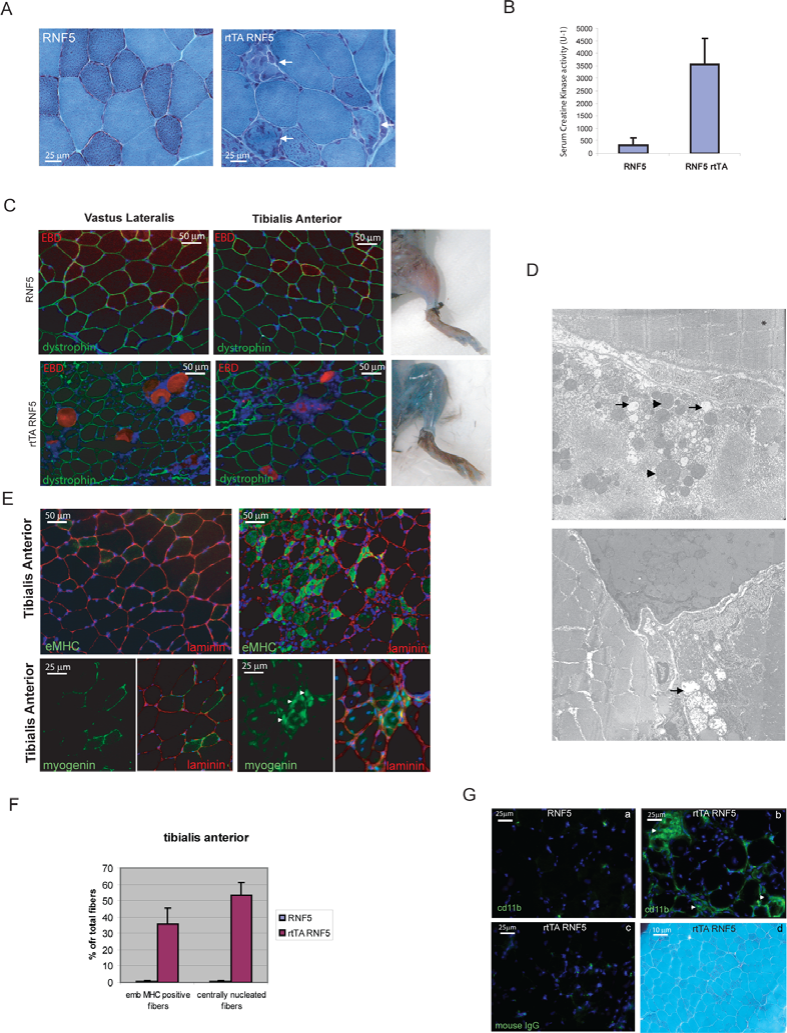
RNF5 induced myodegeneration is associated with extensive myofiber regeneration. A. Modified Gomori-trichrome staining of triceps brachii cross-sections from treated *STg* and *DTg* animals. *DTg* muscle sections show a number of degenerative fibers with cellular infiltration (arrows). B. Serum creatine kinase (CK) levels were monitored in *RNF5 STg* and *rtTA RNF5 DTg* after 4 wk of doxycyclin treatment (n = 3). *DTg* animals had variably elevated CK levels. C. Dye-permeable fibers are surrounded or invaded by macrophagic inflammatory cells clearing necrotic debris. Treated *DTg* and control mice were injected with Evans Blue Dye (EBD) 12 hr before sacrificed and fiber permeability to the dye was monitored externally by the blue coloration of the muscles (right panel) and after cross-section (red fluorescence; left panel). The muscle cross-sections were counterstained with an antibody against dystrophin to visualize fiber boundaries and to highlight degenerative myofibers where staining for dystrophin was absent. D. Ultrastructural analysis of *DTg* muscle sections. In longitudinal sections, sarcomere structure was normal in non-degenerating myofibers (left, asterisks). In an adjacent degenerating fiber, disruption of sarcomere structure, dilated tubular structures (arrows), and enlarged mitochondria (arrowheads) are evident. A similar pattern was evident in cross-section (right panel). Magnification×11,000. E. Muscle cross-sections of doxycyclin-treated *DTg* and control animals stained with antibodies against embryonic myosin heavy chain (embMHC) and myogenin. Arrowheads indicate some nuclei exhibiting a positive myogenin staining in sections from *DTg* animals. Muscle sections were counterstained with anti-laminin α3 antibody (red) and DAPI (blue) to visualize respectively myofiber boundaries and nuclei. F. Quantification of the number of regenerative myofibers, expressed as the average number of embMHC positive fibers (early regenerative fibers) and of centrally nucleated fibers. G. Mice overexpressing RNF5 have extensive mononuclear cell infiltration in areas of myofiber degeneration. Cd11b immunostaining (arrowheads) highlight macrophagic infiltration of triceps brachii muscle cross-sections from *rtTA RNF5 DTg* (b) but not *RNF5 STg* (a) animals treated with doxycyclin for 6 wk. Nuclei are stained with DAPI (blue). Negative control (mouse IgG) performed on sections from *rtTA RNF5 DTg* is shown in panel (c). (d) Muscle regeneration associated with RNF5 overexpression does not result in fibrosis. Triceps cross-sections from RNF5 STg and DTg animals were stained with modified Gomori-Trichrome.

Although vacuolated fibers were not observed, *rtTA RNF5 DTg* animals exhibit myodegeneration, supported by various stages of myonecrosis and phagocytosis based on modified Gomori-trichrome stain (Fig. 4A). Levels of serum creatine kinase (CK) activity were consistently elevated after 6 wk of doxycyclin treatment in the *rtTA RNF5 DTg* animals, but not in their control littermates (Fig. 4B), supporting the presence of myofiber damage. To visualize the extent and distribution of myodegeneration, Evans Blue dye (EBD) was injected into the *rtTA RNF5 DTg* and their control littermates. Contrary to the skeletal muscle of their matching controls, the *rtTA RNF5 DTg* animals exhibited positive staining for EBD within a number of muscle fibers (Fig. 4C), consistent with degeneration (myonecrosis) and elevated serum CK activity. EBD-positive muscle fibers were negative for dystrophin staining (Fig. 4C) and surrounded by immune cells that showed positive staining for the pan-leukocyte marker CD45 and macrophage marker CD11b (data not shown), corresponding to macrophages clearing up necrotic debris. Ultrastructural analysis on *DTg* muscles further confirmed that the sarcomeric structure was conserved in non-degenerative fibers (Fig. 4D asterisk). In degenerating fibers, there was loss of the normal myofibrillar pattern, mitochondrial swelling and dilated tubular structures (Fig. 4D).

Myodegeneration in *RNF5* overexpressing animals was associated with extensive fiber regeneration. Quantitative analysis revealed that 50% of the muscle fibers of *rtTA RNF5 DTg*, but not control animals, were centrally nucleated, and that 30% of the small fibers stained positively for embryonic myosin heavy chain (emb MHC), a known marker of early muscle regeneration (Fig. 4E,F). Consistent with this observation, positive staining for myogenin, a transcription factor expressed during differentiation of activated satellite cells[26], was also observed in small myofibers of *rtTA RNF5 DTg* animal muscle but not in their control littermates (Fig. 4E). Interestingly, clusters of mononuclear cells, staining positively with antibodies against CD45 (data nor shown) and CD11b (Fig. 4G), were also observed at sites of muscle regeneration, yet, neither lymphocytic infiltration nor fibrosis were observed (Fig. 4G, and data not shown). These data indicate that regeneration following overexpression of RNF5 is a result of myofiber degeneration and not an inflammatory process.

Immunostaining using markers for DGC proteins including dystrophin and alpha-sarcoglycan, as well as the extracellular matrix protein laminin α2, did not show any major difference between *DTg* and control muscles (Fig. S3), indicating that disruption of the DGC or extracellular matrix proteins was not a primary cause for myonecrosis. Consistently, *in vitro* differentiation of primary myoblasts cultured from the double transgenic animals treated with doxycyclin prior and during differentiation did not reveal changes in the sarcomeric structure as depicted by staining for alpha-sarcomeric actinin (Fig. S4). Taken together, these findings demonstrate that myofiber degeneration and necrosis account for the elevated serum CK activity associated with RNF5 overexpression, and that myodegeneration is not a primary inflammatory process nor is it a consequence of the alteration of common sarcolemmal structural proteins.

### Myofiber degeneration-coupled regeneration in rtTA RNF5 DTg mice is associated with altered expression of ER chaperones without the formation of protein aggregates

**Figure 5:**
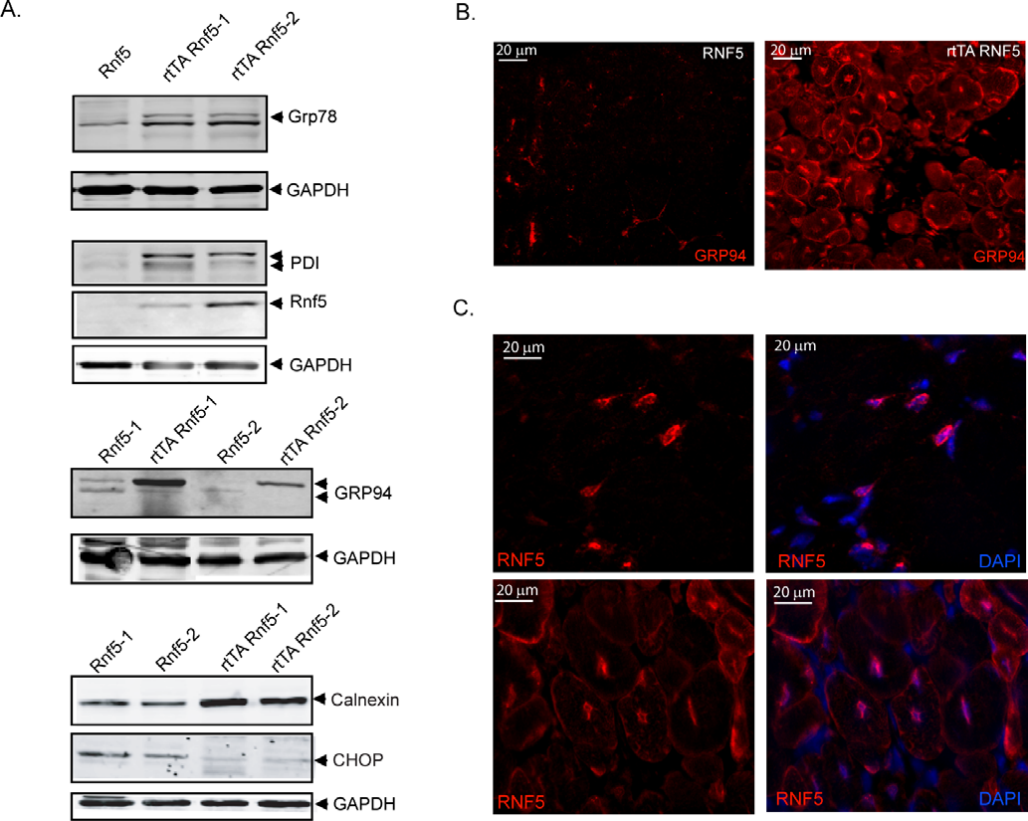
RNF5 localizes to the ER and its overexpression correlates with altered ER function. A. Expression levels of select ER stress markers in muscles of *DTg* animals are elevated compared to their control littermates. Western blot analysis of muscle extract (80 µg) from *RNF5 STg* or *rtTA RNF5 DTg* animals treated for 6 weeks with doxycyclin was probed with Grp78, PDI, Grp94, calnexin and CHOP antibodies. RNF5 and GAPDH antibodies were used for RNF5 expression and loading controls, respectively. B. Grp94 immunostaining of vastus lateralis cross-sections from treated rtTA RNF5 *DTg* and *STg* animals. C. RNF5 localizes to the ER of muscle fibers. Vastus lateralis cross-sections from treated *DTg* animals were stained with RNF5 antibody. Normal (top panels) or regenerative fibers (bottom panels) are shown for RNF5 staining alone (left panels) or combined with DAPI (right panels).

Given the link between ER stress and sIBM [16], [17] we next examined the status of ER stress markers in the muscles of RNF5 overexpressing animals. Consistent with the increase in ER stress markers observed in s-IBM muscles, a clear and consistent, albeit moderate, increase in expression of PDI, Grp78, Grp94 and calnexin was seen in *rtTA RNF5 DTg* but not control mice (Fig. 5A), suggesting that ER stress occurs in the muscles of RNF5 overexpressing animals. Grp94 upregulation was confirmed by immunohistochemistry of the *DTg* muscle sections (Fig. 5B). However, contrary to what has been observed in s-IBM patients, this ER chaperone did not localize to aggregates (Fig. 5B) and congophilic material was not detected in the muscle fibers of *DTg* animals (data not shown). These data suggest that the myodegeneration observed following RNF5 overexpression is associated with ER stress yet occurs at earlier stage in the development of this disease, prior to aggregate formation.

ER stress is commonly considered a protective response. However, high levels of ER stress or ER dysfunction may also impair cell survival by triggering apoptotic pathways (review by [27]. Therefore, the onset of ER stress could cause fiber death with concomitant induction of muscle regeneration without directly affecting the muscle structural components [28], [29], [30]. Analysis of TUNEL and cleaved caspase 3 as markers of apoptosis did not reveal any changes in programmed cell death (data not shown). Similarly, no increase was observed in the levels of CHOP or in cleavage of caspase 12 (data not shown). Rather CHOP levels were decreased in *DTg* animals (Fig. 5A), implying that overexpression of RNF5 may induce ER stress by limiting the activation of some UPR components. These data also indicate that the degeneration process observed in *rtTA RNF5 DTg* animals is not linked with ER-associated programmed cell death.

Grp94 was previously reported to be important for myoblast fusion [31], a critical step in regenerating muscle. Change in Grp94 levels and localization is expected to impact its contribution to myoblast fusion and therefore affect the regeneration process [30]. Analysis of GRP94 expression revealed increased expression and possible post translational modification in *rtTA RNF5 DTg* but not in their matching controls (Fig. 5A). Further, RNF5 staining exhibited dense perinuclear staining in the endoplasmic reticulum of mature and regenerative fibers of the transgenic mice (Fig. 5C). Notably, RNF5 staining was higher in regenerating fibers and localized along the nascent sarcoplasmic reticulum network and sarcolemma (Fig. 5C), suggesting that its function may prevail during the regeneration process. These findings suggest that RNF5 may exert its effect on muscle physiology by modulating the function/localization of specific components of the ER, including Grp94.

### Long-term muscle-specific overexpression of RNF5 results in muscle degeneration and regeneration and vacuole formation

**Figure 6:**
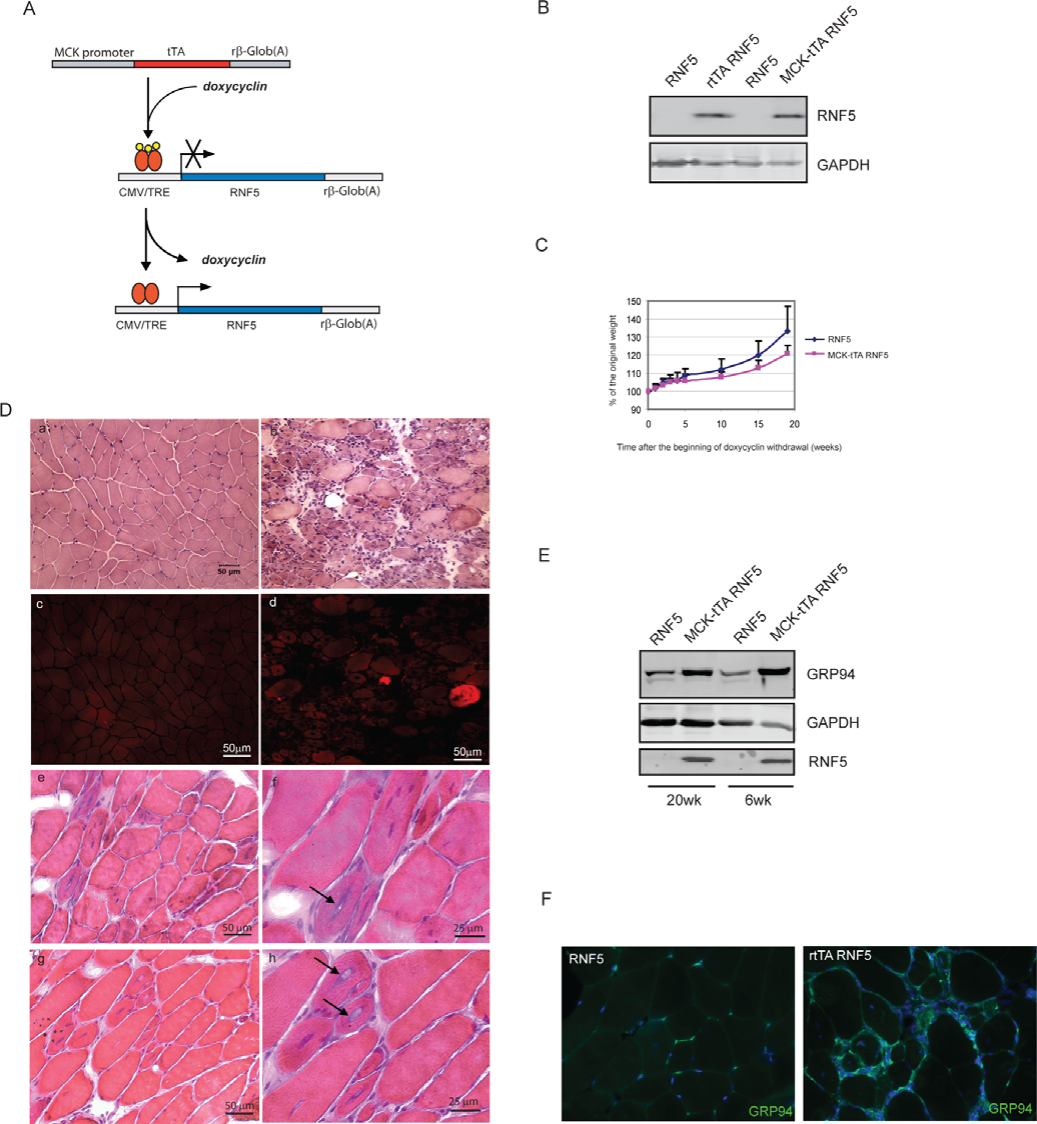
Long term muscle specific expression of RNF5 results in the formation of vacuoles containing aggregates. A. Schematics depicting transgenic constructs used to overexpress *RNF5* in a muscle specific manner. The *tTA* transcriptional activator is expressed under the control of the Muscle Creatine Kinase (MCK) promoter and is inactive in the presence of doxycyclin. Once doxycyclin is withdrawn, tTA activates the expression of the *RNF5* transgene. B. Muscle specific expression of *RNF5* transgene in *MCK-tTA RNF5 DTg* transgenic animals. RNF5 protein levels in vastus lateralis from *rtTA* and *MCK-tTA RNF5 DTg* and *RNF5 STg* mice were monitored by western blot, 20 wk after doxycyclin withdrawal. C. Weight curve comparison of MCK-tTA RNF5-overexpressing *DTg* animals and their matching controls after doxycyclin withdrawal. The body mass of individual animals was monitored for 20 wk. Graphs represent the change in body mass relative to the original weight of a single animal (n = 5). D. Cross-sections of fresh frozen specimens of vastus lateralis muscles from *RNF5 STg (a, c, e, g)* or *MCK-tTA RNF5 DTg* (b, d, f, h) animals 20 wk after doxycyclin withdrawal. H&E (a, b, e, f) and congo red (c, d) stain have been performed on triceps cross-section. Panels f and g represent a higher magnification to better visualize the presence of vacuole containing aggregates (arrows). E. Expression of GRP94 in muscles of *MCK-tTA RNF5 DTg* animals compared to their control littermates, 6 wk and 20 wk after doxycyclin withdrawal. F. GRP94 immunostaining of vastus lateralis cross-sections from MCK-tTA RNF5 *DTg* and *STg* animals 20 wk after doxycyclin withdrawal.

In order to analyze the effect of RNF5 overexpression in a tissue specific manner and to confirm that the phenotype observed originates from RNF5 expression in mature muscles, we crossed RNF5 transgenic animals with mice expressing the *tTA* transgene under the control of the muscle specific promoter MCK (Muscle Creatine Kinase) (Fig. 6A). This mouse model was previously generated to control conditional gene expression in skeletal muscles in a tetracyclin repressible manner [32]. In this model, double transgenic animals (*MCK-tTA RNF5 DTg*) and their matching controls (*RNF5 STg*) were kept under doxycyclin treatment until they reached 3 months of age to prevent the induction of the *RNF5* transgene. Specific overexpression of RNF5 in skeletal muscle was then induced following doxycylin withdrawal. RNF5 level of expression was comparable to the one we observed using β-actin promoter (Fig. 6B). When expressed from a muscle-specific promoter, RNF5 overexpression led to a less severe, albeit clear and reproducible clinical phenotype, with animals surviving up to 5 month after initiating transgene induction. *MCK-tTA RNF5 DTg* were found to exhibit a mild weight loss over time, compared to *STg* (Fig. 6C). This milder phenotype enabled us to monitor the effect of RNF5 overexpression over a prolonged time period. While *MCK-tTA RNF5 DTg* animals exhibited histopathological changes similar to those observed in the β-actin expressing RNF5 mice, only few animals exhibited these phenotype within the same time frame seen for *rtTA RNF5 DTg* mice (6 wk). The majority of *MCK-tTA RNF5 DTg* mice exhibit a clear muscle phenotype only 20 wk after doxycyclin withdrawal (Fig. 6D panels a,b). Strikingly, in addition to the classical myodegeneration and regeneration process previously observed in the *rtTA RNF5 DTg* model, prolonged expression of RNF5 in the *MCK-tTA RNF5 DTg* also caused the appearance of Congo Red positive fibers (Fig. 6D panels c,d) and the formation of blue-rimmed vacuoles containing inclusions (Fig. 6D panels e–h). These findings establish that muscle-specific overexpression of RNF5 is sufficient to cause muscle degeneration with extensive muscle regeneration. Moreover, prolonged RNF5 overexpression leads to the formation of vacuole containing inclusions and accumulation of congophilic material, as seen in sIBM patients, further substantiating a role for RNF5 in the development of the disease.

Similar to what we have observed in *rtTA RNF5 DTg* mice, ER stress markers such as GRP94 was up regulated (possibly a post-translationally modified) in *MCK-tTA RNF5 DTg* animals (Fig. 6E) and elevated expression was observed in small regenerating fibers by immunohistochemistry (Fig. 6F). The two mouse models used provide important confirmation for the role of RNF5 in ER stress-associated muscle degeneration phenotype.

### RNF5 KO mice exhibit a delay in regeneration from cardiotoxin-induced muscle damage

**Figure 7:**
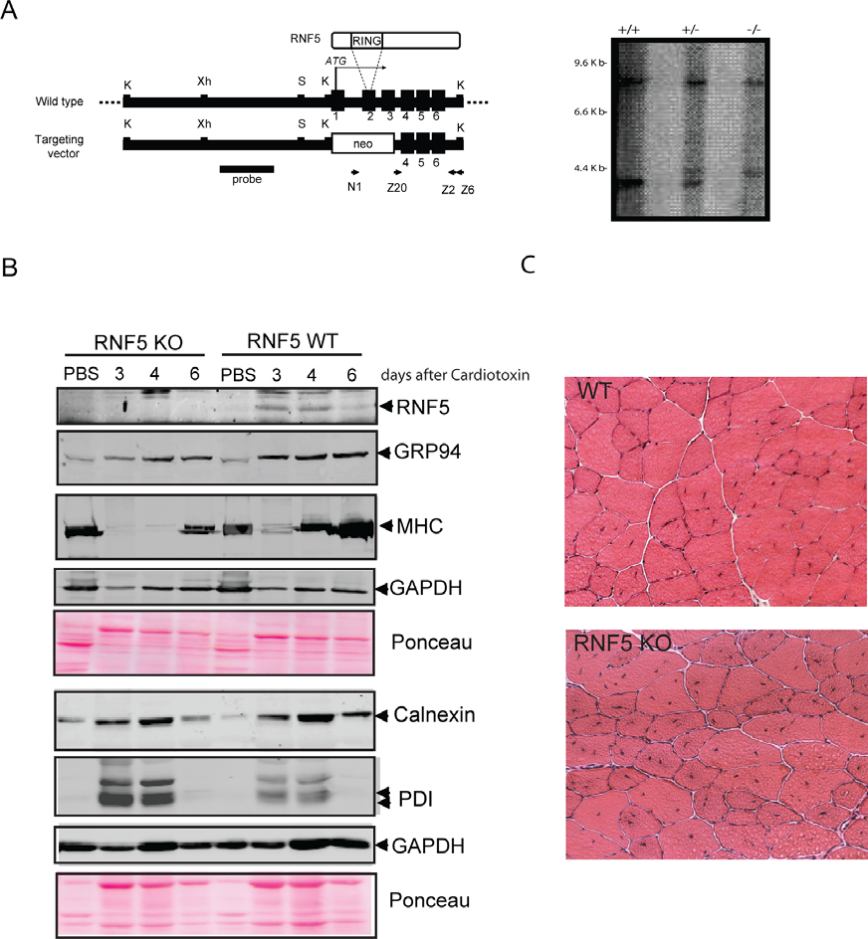
Delayed Muscle regeneration in RNF5 KO mouse subjected to muscle damage by cardiotoxin treatment. A. Generation of RNF5 KO mouse. Schematic of RNF5 genomic structure (upper) and targeting vector (lower) are shown. Restriction enzyme sites (K: KpnI, Xh: XhoI, S: SalI) are marked. Location of the primers for PCR and the probe for Southern blotting are indicated. Exons 1, 2 and 3 including the translational start site (ATG) and RING finger domain are substituted with neo cassette. Southern blot for analysis of RNF5 gene is shown on the right panel. DNA isolated from the tails of +/+, +/−, and −/− mice was digested with EcoRI and resolved by electrophoresis through 0.8% agarose gels. After transferring to nitrocellulose paper, blots were hybridized with radiolabeled cDNA probes corresponding to the fragment indicated in diagram. B. Protein extracts from cardiotoxin and PBS treated tibialis muscles from WT and RNF5 KO animals were analyzed by western blots with the indicated antibodies to monitor changes in endogenous RNF5 as well as ER stress and muscle markers. Ponceau staining is used as a loading control. C. Cross section of tibialis anterior from WT and RNF5 KO mice performed 28 days after cardiotoxin treatment.

To further assess the potential role of RNF5 in normal muscle physiology we used an RNF5 KO model established in our laboratory. In these mice, exons 1-3 of the *RNF5* gene were replaced by a Neo cassette (Fig. 7A). The chimeric mice gave a germline transmission of the disrupted *RNF5* gene (see Materials and Methods for further details) and heterozygous mice were interbred to produce litters that included homozygous offspring. Southern blot analysis of mouse DNAs isolated from the tails of these mice revealed the expected three genotypes (+/+, +/−, −/−), as judged by diagnostic probes for the correct 5′ and 3′ homologous recombination events (Fig. 7A).

*RNF5* KO and WT animals were subjected to cardiotoxin treatment, which induces muscle damage followed by its regeneration [33], thereby allowing us to monitor possible changes in the ability of each genotype to promote muscle regeneration. The toxin, or PBS control, was injected directly into the tibialis muscle and the muscles were collected after 3, 4, 6, 12 and 28 days.

Significantly, the expression of RNF5 in WT muscle was elevated after cardiotoxin treatment (Fig. 7B), thereby supporting a role for RNF5 in muscle physiology/regeneration. To assess whether increase of RNF5 levels in physiological conditions would affect ER stress markers, we monitored the levels of GRP94 in RNF5 KO and WT during the course of muscle regeneration. Importantly, both induction of Grp94 expression and its post translational modification, as defined by the disappearance of the slower migrating form, were delayed in RNF5 KO mice, compared with WT animals (Fig. 7B and Fig. S5). Of interest, induction of calnexin was slightly delayed whereas expression of PDI was elevated in RNF5 KO.

Consistent with the changes observed in induction of ER stress markers, muscle regeneration in RNF5 KO mice was attenuated, compared to control animals, as evident from (i) delayed production of myosin heavy chain (Fig. 7B) and (ii) a high number of centrally nucleated fibers observed 28 days after cardiotoxin treatment in the KO but not WT animals (Fig. 7C). These data substantiate the observations made with RNF5 Tg mice and suggest that *RNF5* is playing an important role in muscle regeneration process associated with ER stress response.

## Discussion

The present study establishes a link between RNF5 and specific muscular disorders associated with ER stress. Prompted by our observation that RNF5 is overexpressed and mislocalized to aggregates in muscle biopsies from sIBM patients and deregulated in an animal model for hereditary IBM, we have investigated the role of RNF5 in muscle using 3 genetic mouse models: an inducible transgenic mouse in which the E3 ligase RNF5 is conditionally overexpressed (globally and selectively in skeletal muscle) and an RNF5 KO mouse. RNF5 overexpression led to myodegeneration and regeneration in skeletal muscle independent of the classical dystrophic mechanism of sarcomeric disruption. In addition, prolonged RNF5 overexpression leads to the formation of blue-rimmed vacuoles containing inclusions and congo red positive fibers, as seen in sIBM. Further, RNF5 KO mice subjected to muscle damage exhibited a milder muscle phenotype which is consistent with a slower regenerative process. Both RNF5 overexpression and KO models suggest that the muscle phenotypes are associated with altered ER stress response, as shown by the increase in the ER stress markers PDI, GRP78, GRP94 and calnexin in the muscle of the RNF5-overexpressing animals and the delay in GRP94 and calnexin induction in RNF5 KO. These observations establish a link between RNF5, ER stress and muscle physiology. The mouse lines established and characterized in this study offer new models to study ER-related myodegenerative process that occur in myopathies such as sporadic or hereditary IBM.

Impairment of the ERAD and UPR pathways has been linked with the onset of degenerative diseases such as sIBM, and is thought to have a contributing role by allowing improper accumulation of malfolded proteins. Consistent with this hypothesis, we found that RNF5, an E3 ligase involved in ERAD, exhibit an altered pattern of expression in biopsies from sIBM patients. Interestingly, RNF5 has also been shown to be upregulated in brain (Substantia Nigra) from Parkinson Disease patients [34], therefore pointing to a possible broader role of this E3 ligase in degenerative disorders. The increase in RNF5 expression seen in these degenerative diseases could be a consequence of ER overload, ERAD dysfunction, or part of the mechanism engaged in the development of these disorders. Our data supports the latter possibility since RNF5 overexpression is sufficient to induce the myodegenerative process associated with both the formation of blue-rimmed vacuole over time and an elevation of ER stress markers, characteristic of sIBM. Our observation that RNF5 over-expression was not sufficient to promote the formation of vacuoles containing inclusions at an early time point when ER stress markers were already upregulated suggests that ER stress and RNF5 overexpression are among early events in the pathology of these muscle disorders.

That RNF5 is deregulated in the DMRV mouse model, where a sialation defect has previously been shown to promote the IBM-like phenotype, implies that RNF5 deregulation may not be the primary cause for the disease but rather be one of the early components contributing to the pathological process.

Among mechanisms that may account for the ability of RNF5 to cause myodegeneration is the effect of RNF5 on ER stress response and ERAD. RNF5 contributes to ubiquitination of misfolded proteins, with a concomitant effect on their clearance of the proteins by the proteasomes [23]. RNF5 also controls ERAD through its effect on JAMP, an ER anchored 7 transmembrane protein which is important for proteasome recruitment to the ER (our unpublished observation). Thus, RNF5 overexpression is expected to impair the ER stress response, consistent with the modification of ER stress associated chaperones observed in our mouse model. Of importance is that accumulating pathological evidence now links mutations or dysregulation of ER-related proteins with muscular disorders. For example, mutations in the ER-associated ATPase p97/VCP have been associated with a specific type of inclusion body myopathy associated with Paget disease [35]. In addition, the p97/VCP and ERAD pathways were recently implicated in the degradation of sarcomeric myosin and its chaperone Unc-45 [36], [37], thereby offering a link between physiologic ER function and the organization of essential structural muscle components. Further, activation of ER stress was implicated in an animal model of autoimmune myositis with increased expression of MHC class I in muscle fibers and in human myositis patients [38]. ER overload may be sufficient to trigger muscle regeneration prior to, and independently of, the onset of inflammation, supporting ER dysfunction as a primary cause of fiber damage [38], [39]. Thus, RNF5 overexpression may impair the ER function, ultimately leading to a defect in the process of muscle maintenance.

Independent evidence also supports the fact that ER-associated processes are physiologically relevant during muscle regeneration. The chaperone protein GRP94 and the ER-stress-responsive transcription factor ATF6 were upregulated and required for proper myoblast differentiation *in vitro* [29], [31]. UPR has recently been proposed to be part of a quality control mechanism that determines proper muscle differentiation. In this model, the induction of certain UPR components, such as the transcription factor Xbp1, is used as a threshold to determine the progression of the myoblast into the differentiation program and the survival of the differentiated fiber [40], [41], [42]. Deregulation of RNF5 in this context is expected to interfere with the balance of these components most probably as a downstream component of this system, therefore altering the differentiation and the muscle maintenance programs. Of interest, RNF5 E3 ligase activity does not appear to be required for the muscular phenotype, as Tg mice that expresses RING mutant form of RNF5 exhibits similar phenotypes (data not shown). Consistent with this observation, a close homolog of RNF5, RNF185, was shown to control paxillin turn-over during Xenopus development independently of its E3 ligase activity [43]. Among possibilities to explain ubiquitin ligase independent effect of RNF5 is that RNF5 may impair muscle function by squelching or recruiting components of the ER machinery, by outcompeting its associated ligases (i.e. CHIP or gp78), or by acting as an E4 ligase on specific substrates after their ubiquitination by another E3 for which ligase activity may not always be required (i.e. p300 [44]).

In conclusion, the characterization of the RNF5 transgenic and KO mouse models establishes an undisclosed link between impaired ER stress response and the early changes in degenerative myopathies, shown here for sIBM, while offering a novel model for studying the complex function of the ER in global muscle physiology.

## Methods

### Generation of *RNF5* transgenic mice

Mouse studies were performed under IACUC approvals from MSSM and BIMR. The mouse isoform of the *RNF5* gene was cloned by PCR in frame with the HA tag into the pTRE2-HA vector using MluI and NheI restriction sites and sequenced. The linear fragment resulting from a HpaI-SapI digestion was then used for pronuclear injection. After microinjection into B6C3 (C57BL/6×C3H) F2 hybrid eggs, the fertilized eggs were transferred into C57/BL6 female recipients and crossed with C57/BL6 males. Conditional *RNF5* overexpression was achieved by crossing *RNF5 STg* animals with *rtTA Tg* mice, expressing the tetracycline-responsive Transcriptional Activator under control of the ubiquitous CMV-β-actin promoter, and the genotypes were verified by PCR reaction using the following primers (RNF5-forward:

1. GTACCCATACGATGTTCCAGATTACGC;
2. RNF5-reverse: CTGAGCAGCCAGAAAAAGAAAAAGATG;
3. rtTA-forward: CGGGTCTACCATCGAGGGCCTGCT;
4. rtTA-reverse: CCCGGGGAATCCCCGTCCCCCAAC);

Both *RNF5* and *rtTA* transgenic lines were kept as heterozygous and maintained as separate lines by crossing with WT C57/BL6 animals.

MCK-tTA transgenic animals were obtained from Taconic Farms, as a repository for NIH deposited mouse strains and genotypes using the same primers as for rtTA animals.

### Immunoblot and immunohistochemistry analysis

For expression analysis, frozen tissues were collected, flash frozen and pulverized using a mortar and pestle in liquid nitrogen. Proteins were extracted by resuspension in cold RIPA buffer containing anti-proteases and the extracts homogenized and clarified by high speed centrifugation. The protein concentration in the supernatant was determined by Bradford assay. RNF5 expression was analyzed either by straight immunoblot or after immunoprecipation using an affinity-purified RNF5 polyclonal antibody (1:2000 dilution). Expression for ER stress markers was assessed using rabbit GRP78 (Santa Cruz), rabbit GRP94 (Abcam) and mouse PDI (StressGene) antibodies and using GAPDH (Ambion) as a control.

For immunohistochemistry analysis, skeletal muscle tissues embedded in O.C.T. Compound (Sakura Tissue-Tek, product #4583) were pinned on cork pieces and snap-frozen in isopentane cooled in liquid nitrogen. Frozen tissues were stored at −80°C until cross-sections (8 µm thick) were prepared using a Leica CM3050S cryostat and stained.

H&E stainings and Gomori-Trichrome were performed as previously described [45]. For immunostaining, sections were fixed in cold acetone for 5 min, permeabilized with 0.1% Triton X for 10 min and blocked with 1% glycine for 30 min. Immunostainings were performed using dystrophin antibody (Vector clone Dy8/6C5, diluted 1:20), CD45 (BD Pharmingen, clone 30-F11, diluted 1∶200), Cd11b (BD Pharmingen, clone M170, diluted 1∶100), embMHC (hybridoma bank F1.652, diluted 1∶3), myogenin (hybridoma bank F5D, diluted 1∶100), laminin α2 antibody (abcam 4H8-2,). RNF5 polyclonal antibody generated using full length bacterially produced RNF5. Rabbit's serum was purified on RNF5 affinity column and used (dilution 1∶100). For mouse monoclonal antibodies, the sections were incubated in biotinylated anti-mouse and avidin-conjugated fluorescein. (Mouse on Mouse Kit, Vector #FMK2201) For rat and rabbit antibodies, the sections were incubated with alexa fluor conjugated secondary antibodies and diluted 1∶600 in Dako antibody diluent (#S3022) for 1 h at RT. Images were captured using an Olympus IX71 fluorescence microscope and the Slidebook version 4.0 software.

Samples from human muscular disorders were obtained under IRB approval at the corresponding collaborating institutes in Italy and Japan. For the human sample processing, immunohistochemistry was performed on unfixed cryostat sections of diagnostic muscle biopsies from patients with the following diagnosis: s-IBM (10), DMD (2), BMD (2), normal muscle (3). After blocking, sections were incubated overnight at 4° with RNF5 antibody (1∶100) and either monoclonal anti-phosphorylated tau SMI31 (1∶5000, (Sternberger Monoclonals, Baltimore, MD, USA) or anti-beta amyloid protein (1∶100, Biosource International, Camarillo, CA, USA, clone 6E10). Sections were incubated for 1 hr at RT with the appropriate Alexa fluor-conjugated secondary antibodies and Hoechst 33258 (Molecular Probes Inc.) staining was used to visualize cell nuclei. Slides were analyzed under a laser scanning confocal microscope SP5 (Leica, Germany)

### Phenotypic analysis of double transgenic and control animals

A 2 mg/ml solution of doxycyclin supplemented with 5% sucrose was given to littermates animals between 12 wk and 24 wk of age in drinking water for 6 wk. Phenotypic alteration (body mass, weakness, blood withdrawal) were observed bi-weekly and the animals were sacrificed after 6 wk of treatment. Organs were individually weighed on a precision sale after trimming of the extra-tissues. Organs were then flash frozen in liquid nitrogen for expression analysis and frozen in OTC for immunohistochemical analysis.

### Electron Microscopy

Glutaraldehyde-fixed muscle specimens were post-fixed in osmium tetroxide, and dehydrated in serial alcohol solutions and propylene oxide prior to embedding in araldite resin. Thick sections (1 µm) were stained with toluidine blue for light microscopy and ultrathin (60 nm) sections stained with uranyl acetate and lead citrate for electron microscopy.

### Morphometric analysis of skeletal muscle cross-sections

Cross-sections of the triceps brachii, tibialis anterior, and vastus lateralis muscles from the double transgenic animals and their matching controls were immunostained with dystrophin and H&E to delineate the fibers, and the cross-section area of each fiber was quantified using Scion software (version 4.0.3.2; NIH).

### Evans Blue Dye Injection

Evans Blue Dye (SIGMA) was diluted in PBS (10mg/ml) and filter-sterilized and the dye was injected through the tail vein at a concentration of 100µl per gram of body weight. Sixteen hours after injection, the mice were sacrificed and dissected muscles inspected for presence of blue dye in the muscle. Skeletal muscles were then fresh-frozen and cross-sections were further analyzed for presence of dye and counter-stained with dystrophin antibody as described above.

### Serum Creatine Kinase Assay

150 µl of blood was collected in heparin-treated tubes from periorbital bleeding and the serum fraction extracted by double centrifugation for 5 min at 5000 rpm. Creatine kinase levels were monitored using CPK-NAC kit (JAS Diagnostics #CPK1-15) and analyzed on a Roche Cobas Mira classic apparatus.

### Isolation of primary myoblast from muscle fibers

One month old animals (*RNF5 STg* and *rtTA RNF5 DTg*) were sacrificed and tibialis anterior, gastrocnemius and soleus muscles were collected and rinsed in sterile PBS. Muscles were then digested in sterile filtered type 1 collagenase (0.2%) in DMEM using a shaking water bath for 2 hours at 37 degrees. Muscle fibers were then extracted by trituration after dilution of the digestion medium in one volume of DMEM. Single muscle fibers were then separated from debris by gently aspirating the fibers under a dissecting microscope and transferring them in a new dish containing DMEM plus 10% horse serum. This step was repeated 3 or 4 times in a row to separate intact fibers from the debris. 20 to 30 fibers were then plated in 60 mm dishes coated with 10% Matrigel and were incubated for 12–24 hours in DMEM plus 10% horse serum. Satellite cells were then allowed to proliferate for 3 days by changing the medium to DMEM plus 20% FBS, 10% horse serum, 0,5% chick embryo extract. Differentiation of myoblasts was subsequently achieved by changing the medium to 2% horse serum plus 0.5% chick embryo extract in DMEM. Doxycyclin (2 µg/ml) was added to the proliferation medium and the differentiation media to promote and maintain the expression of RNF5 transgene before and during the differentiation process.

### Immunocytochemistry

Cells were washed in PBS and fixed using freshly prepared 3% paraformaldehyde in PBS (10 min at room temperature). The cells were then washed in PBS, followed by permeabilization in 0.1% Triton X-100 in PBS (pH 7.4) for 5min on ice and an additional three washes in PBS. Cells were then incubated in PBS supplemented with 3% bovine serum albumin for 30 min. The cells were incubated with antibodies (1 h at room temperature) in a humidity chamber. The cells were washed in PBS (3×, 5 min each) before incubation with 100-µl of Alexa-488- and Alexa-568-conjugated anti-rabbit or anti-mouse immunoglobulin G (Molecular Probes) diluted (2 µg/ml) in PBS containing 3% BSA (60 min at room temperature in a light protected humidity chamber). The cells were rinsed three times in PBS and mounted on glass slides using Vectashield (Vector Laboratories). Primary antibodies were used as follows: RNF5 (1∶200), sarcomeric alpha-actinin (Sigma, 1∶5000), Pax7 (Hybridoma Bank, 1∶10).

### Generation of RNF5 KO animals

RNF5 targeting vector consisted of a 9kb 5′ homologous region and 1.5 kb 3′ homologous region of RNF5 genomic sequence which was inserted into 5′ of Neo gene cassette using PspOMI site. The Neo gene cassette was inserted to replace exon 1, 2 and 3 including the first ATG. Linearized targeting vector (10 µg) was transfected by electroporation into 129SvEv ES cells and screened for G418-resistant clones. Surviving colonies were expanded and PCR analysis was performed to identify clones that had undergone homologous recombination. Homologous recombination was confirmed by PCR using primer pairs, ZESA6 5′-GCCAGCTGAAGGTGAGGGACTGGAC-3′ and Neo1 5′-TGCGAGGCCAGAGGCCACTTGTGTAGC-3′ The correctly targeted ES cell lines were microinjected into C57BL/6J blastocytes. The chimeric mice were generated and selected for a germline transmission of the disrupted RNF5 gene. Heterozygous mice were interbred to produce litters that included homozygous offspring. Additional analysis was carried out using DNA prepared from the MEFs that were obtained from these mice, further confirming the deletion of RNF5 gene. Chimeric mice with germline transmission were mated with C57BL/6J. The targeting vector and KO mice were generated by ITL, (Long Island NY). Backcross to C57BL/6J allowed to generate >90% strain. Genotyping were carried out by Southern blotting or PCR. Primers used for PCR were ZESA2 5′-CCCTATGTCCTACAGGCTCTG-3′ and ZESA20 5′-ACACGATGCTGAGGGGAGCTGCAG-3′ for the wild type allele, and ZESA6 and Neo1 for the targeted allele.

### Cardiotoxin treatment

Four-month-old wild-type and RNF5 null animals were anaesthetized with isoflurane, and 100 µl of 10 µM cardiotoxin (CalBiochem) in 1xPBS was injected into the right tibialis muscle. The animals were sacrificed at 3, 4, 6, 12 and 28 days after cardiotoxin injection. Tibialis muscles were harvested tendon-to-tendon and mounted in OCT or flash frozen in liquid nitrogen for histological or protein analysis.

### *RNF5* silencing by siRNA

The pCMS3-cherry vector was used to transfect short interfering RNA (siRNA) targeting *RNF5* and its corresponding scramble control into Hela cells. Two 64-base complementary oligonucleotides (5′GATCCCCAGCTGGGATCAGCAGAGAGttcaagagaCTCTCTGCTGATCCCAGCT TTTTTGGAAA3′ and 5′AGCTTTTCCAAAAAAGCTGGGATCAGCAG AGAGTCTCTTGAACTCTCTGCTGATCCCAGCTGGG-3′) were synthesized to contain a RNF5 19-nucleotide sequence or its scramble counterpart ( 5′-GATCCCC AGCTGGCATCAGCAGGGAG ttcaagaga CTCCCTGCTGATGCCAGCT TTTTTGGAAA 3′ and 5′AGCTTTTCCAAAAAAGCTGGCATCAGCAGGGA GTCTCTTGAACTCCCTGCTGATGCCAGCTGGG 3′). The annealed product containing 5′ and 3′ overhangs compatible with BglII and HindIII restriction sites, respectively was then ligated into pCMS3-cherry digested with BglII and HindIII [46].

## Supporting Information

Figure S1 Specificity of RNF5 antibody. A. Specificity of RNF5 staining in cells where RNF5 expression has been downregulated by specific shRNA. HeLa cells were transfected with a plasmid expressing both the Cherry fluorescent marker and RNF5-specific shRNA or its scrambled version and analyzed for RNF5 expression by immunocytochemistry after 36 hours. B. Specificity of RNF5 antibody on muscle cross-sections. Triceps brachii cross-sections from RNF5 DTg or STg were stained with RNF5 antibody or a similar concentration of rabbit IgG. Arrowheads point areas of RNF5 staining.
(2.89 MB TIF)

Figure S2 RNF5 expression in human myopathies. RNF5 immunostaining of muscle sections from Duchenne Muscular Dystrophy (DMD) and Becker Muscular Dystrophy (BMD) patients compared to a normal muscle cross-section. Arrowheads point to RNF5 staining.
(4.86 MB TIF)

Figure S3 RNF5 overexpression is not associated with alterations in the dystrophin-glycoprotein complex. Cross-sections of vastus lateralis from treated DTg or control mice were subjected to immunostaining using dystrophin (A) laminin α3 (B) and alpha-dystroglycan (C), revealing a normal pattern of sarcolemmal and extracellular matrix staining.
(4.51 MB TIF)

Figure S4 RNF5 overexpression does not prevent proper differentiation and sarcomeric organization of primary myoblasts. Primary myoblasts were isolated from 1 month old animals (RNF5 STg and rtTA RNF5 DTg) and grown in the presence of doxycyclin (2 µg/ml). Differentiation was achieved by switching cells to differentiation medium supplemented with ITS (Insulin Transferrin Selenium supplement). Induction of RNF5 in primary myoblasts prior to differentiation was confirmed by co-immunostaining with Pax7 (A) and the efficiency of myoblast differentiation was monitored by analysis of phase contrast images (bottom panels) and immunostaining the sarcomeres with sarcomeric alpha-actinin (upper panels) (B).
(9.47 MB TIF)

Figure S5 GRP94 expression in muscles of cardiotoxin-treated RNF5 KO and WT animals. Proteins prepared from muscle of RNF5 KO or WT animals were quantified and analyzed by western blot analysis using antibodies to GRP94. Upper band correspond to a post translationally modified form of GRP94.
(0.45 MB TIF)
